# Fungi and Fungal Metabolites for the Improvement of Human and Animal Life, Nutrition and Health

**DOI:** 10.3390/jof10120863

**Published:** 2024-12-12

**Authors:** Laurent Dufossé

**Affiliations:** 1 Laboratoire de Chimie et Biotechnologie des Produits Naturels—CHEMBIOPRO, Université de la Réunion, 15 Avenue René Cassin, CEDEX 9, CS 92003, F-97744 Saint-Denis, Ile de la Réunion, France; laurent.dufosse@univ-reunion.fr; 2 Ecole Supérieure d’Ingénieurs Réunion Océan Indien—ESIROI, 2 Rue Joseph Wetzell, F-97490 Sainte-Clotilde, Ile de la Réunion, France; 3 Laboratoire ANTiOX, Université de Bretagne Occidentale, F-29000 Quimper, France

Fungi: 1, 2, 3,... 5.1 million species? Even scientists do not currently agree on how many fungi there might be on the planet Earth, but only about 140,000 have been described so far [1]. They have been grouped into a separate kingdom of organisms, as complex and diverse as plants and animals, of which only a small percentage have been named and described. Fungal biomasses and fungal metabolites have a long common history with human and animal life, nutrition, and health. Macro fungi and filamentous fungi have a large portfolio of proteins, lipids, vitamins, minerals, oligo elements, pigments, colorants, bioactive compounds, antibiotics, pharmaceuticals, etc. For example, industrially important enzymes and microbial biomass proteins have been produced from fungi for more than 50 years. Some start-ups convert by-products and side-streams rich in carbohydrates into a protein-rich fungal biomass. The biomass is then processed into a vegan meat substitute for food applications. In the last few years, there has also been a significant rise (in fact, a significant revival) in the number of publications in the international literature dealing with the production of lipids by microbial sources (the “single cell oils; SCOs” that are produced by the so-called “oleaginous” microorganisms, including “oleaginous” fungi, such as the zygomycete species, e.g., *Cunninghamella echinulata* and *Mortierella isabellina*). Fungi are potential sources of polyunsaturated fatty acids (PUFAs), as these microorganisms can accumulate large amounts of high-valued PUFAs, such as gamma-linolenic acid (GLA) and arachidonic acid (ARA).

The objective of the invitation to contribute to this MDPI Fungi Special Issue was not to give complete coverage of how fungi and fungal metabolites are able to improve human and animal life, nutrition, and health. I, as guest editor, simply wanted to encourage authors working in this field to publish their most recent work in a rapidly expanding journal in order to make a large audience discover the full potential of wonderful and beneficial fungi. Thus, this Special Issue welcomes 16 scientific contributions (10 original research papers and six reviews) on the applications of fungi and fungal metabolites, such as bioactive compounds, nephroprotectants, mycoproteins, antifungals, insecticides, antibacterials, enzymes, etc., with great potential in human and animal life, nutrition, and health.

Many original research papers of this Special Issue deal with fungal endophytes. Plants in ecosystems mostly appear to be symbiotic with fungal endophytes. This highly diverse group of fungi can have profound impacts on plant communities through increasing fitness by conferring abiotic and biotic stress tolerance, increasing biomass, and decreasing water consumption, or decreasing fitness by altering resource allocation [2]. Fungal endophytes have remarkable potential to produce a wide range of pharmacologically significant bioactive compounds that are used in disease management and human welfare. In the study by Verma et al. [3], a total of eight fungal endophytes were isolated from the leaf tissue of *Amoora rohituka*. HPTLC fingerprint analysis and antioxidant activity of ethyl acetate extract from isolated *P. oxalicum* revealed that fungal endophytes produce bioactive compounds in a host-dependent manner. More investigations are being conducted in order to have access to the chemical structures that are produced. Very large reviews are useful to newcomers in a research area. Deshmukh et al. [4] reported 451 bioactive metabolites isolated from various groups of endophytic fungi from January 2015 to April 2021, along with their antibacterial profiling, chemical structures, and mode of action. Bioactive metabolites with unique skeletons have been identified, which could be helpful in the prevention of increasing antimicrobial resistance. Antibiotic resistance is becoming a burning issue due to the frequent use of antibiotics to cure common bacterial infections, indicating that we are running out of effective antibiotics. The review by Nagarajan et al. [5] highlights the recent developments in metabolomics studies of endophytic fungi in obtaining a global picture of metabolites. Metabolomics coupled with advanced analytical tools provides a comprehensive insight into systems biology. Despite its wide scientific attention, endophytic fungi metabolomics is still relatively unexploited, and new techniques will increase the number of commercial applications.

Moving to other sources of bioactive metabolites, Meade et al. [6] describe how mushrooms have been used as traditional medicine for millennia. This literature review aims to explore recent evidence relating to the application of fungal bioactives in treating chronic mental health and chronic pain morbidities. There is now increasing interest in using fungal active compounds such as psychedelics for alleviating symptoms of mental health disorders, including major depressive disorder, anxiety, and addiction. Also related to human health, the research conducted by Sinaeve et al. [7] investigates how *Ganoderma* extracts may have nephroprotective effects. Although cisplatin is used as a first-line therapy in many cancers, its nephrotoxicity remains a real problem. Acute kidney injuries induced by cisplatin can cause proximal tubular necrosis, possibly leading to interstitial fibrosis, chronic dysfunction, and finally to the cessation of chemotherapy. The study focused on the respective in vitro effects of methanolic extracts of *Ganoderma tuberculosum* Murill. and *Ganoderma parvigibbosum* Welti & Courtec. and their association with human proximal tubular cells (HK-2) intoxicated by cisplatin. *Fusarium* fungi belong to a large genus of filamentous fungi, part of a group often referred to as hyphomycetes, widely distributed in soil and associated with plants. Rana et al. [8] emphasize that the taxonomy of the genus *Fusarium* has been in a flux because of ambiguous circumscription of species-level identification based on morphotaxonomic criteria. In this study, multigene phylogeny was conducted to resolve the evolutionary relationships of 88 Indian *Fusarium* isolates based on the internal transcribed spacer region, 28S large subunit, translation elongation factor 1-alpha, RNA polymerase second largest subunit, beta-tubulin, and calmodulin gene regions. Additionally, there was a special focus on beauvericin (BEA), and, among the 88 isolates studied, 50 were capable of producing BEA, which varied from 0.01 to 15.82 mg/g of biomass. Antifungal compounds are also very important bioactive metabolites produced by filamentous fungi. The primary purpose of the study by Kuvarina et al. [9] was to isolate and identify a hydrophobin, Sa-HFB1, from an alkaliphilic fungus, *Sodiomyces alkalinus*. The highest level of antifungal activity (MIC 1 µg/mL) was demonstrated for the clinical isolate *Cryptococcus neoformans* 297 m. Fungal metabolites may also target insects. Chemical insecticides can cause significant harm to both terrestrial and aquatic environments. New insecticides derived from microbial sources are a good option with ‘no’ or low environmental consequences. *Metarhizium anisopliae* (mycelia) ethyl acetate extracts were tested in [10] on larvae, pupae, and adults of *Anopheles stephensi* (Liston, 1901), *Aedes aegypti* (Meigen, 1818), and *Culex quinquefasciatus* (Say, 1823), as well as non-target species *Eudrilus eugeniae* (Kinberg, 1867) and *Artemia nauplii* (Linnaeus, 1758). Among numerous bioassay results, *Metarhizium anisopliae* extracts had remarkable toxicity on *Aedes aegypti* and *Culex quinquefasciatus*.

*Erwinia mallotivora*, the causal agent of papaya dieback disease, is a devastating pathogen that has caused a tremendous decrease in Malaysian papaya exports and affected papaya crops in neighboring countries. In the research conducted by Tamizi et al. [11], mycelial suspensions from five rhizospheric *Trichoderma* isolates of Malaysian origin were found to exhibit notable antagonisms against *E. mallotivora* during co-cultivation. Based on these findings, the fungal isolates are proven to be useful as potential biological control agents against *E. mallotivora*, and the genomic data opens possibilities to further explore the underlying molecular mechanisms behind their antimicrobial activity, with potential synthetic biology applications.

Antimicrobial secondary metabolites from *Fusarium oxysporum* R1 associated with the traditional Chinese medicinal plant *Rumex madaio* Makino were screened for many years by Fu et al. [12] using the one strain, many compounds (OSMAC) strategy. Two diastereomeric polyketides, neovasifuranones A and B, were obtained from their solid-rice-cultivated medium together with N-(2-phenylethyl)acetamide, 1-(3-hydroxy-2-methoxyphenyl)-ethanone, and 1,2-seco-trypacidin. Their planar structures were unambiguously determined using 1D NMR and MS spectroscopy techniques as well as comparison with the literature data. The accurate determination of the chemical structures of fungal products is of high priority.

Yeasts producing semiochemicals are increasingly used in pest management programs; however, little is known about which yeasts populate cherry fruits, and no information is available on the volatiles that modify the behavior of cherry pests, including *Rhagoletis cerasi* flies. Eighty-two compounds were extracted by Mozūraitis et al. [13] from the headspaces of eleven yeast species associated with sweet and sour cherry fruits using solid-phase microextraction. Two-choice olfactometric tests revealed that *R. cerasi* flies preferred 3-methylbutyl propionate and 3-methyl-1-butanol but avoided 3-methylbutyl acetate. Yeast-producing behaviorally active compounds indicated a potential for use in pest monitoring and control of *R. cerasi* fruit flies, an economically important pest of cherry fruits.

Another food-related paper deals with the *Issatchenkia terricola* strain WJL-G4, which has great potential in red raspberry wine fermentation [14]. In the current study, *I. terricola* WJL-G4 was applied to decrease the content of citric acid in red raspberry juice, followed by the red raspberry wine preparation by *Saccharomyces cerevisiae* fermentation, aiming to investigate the influence of *I. terricola* WJL-G4 on the physicochemical properties, organic acids, phenolic compounds, and antioxidant activities during red raspberry wine processing.

Fungal enzymes also have a huge impact on human and animal life, nutrition, and health. A comprehensive review by El-Gendi et al. [15] elaborates on the different types and structures of fungal enzymes as well as the current status of the uses of fungal enzymes in various applications. Mycoproteins are currently a hot topic. The main findings by Derbyshire [16] showed that fungal mycoproteins could contribute to an array of health benefits across the animal and human lifespan, including improved lipid profiles, glycaemic markers, dietary fiber intakes, satiety effects, and muscle/myofibrillar protein synthesis. Continued research is needed in human nutrition, which would be worthwhile at both ends of the lifespan spectrum and in specific population subgroups.

Research is also being conducted on the detrimental effects of filamentous fungi, such as those from multidrug-resistant species belonging to the *Scedosporium* genus [17] that are well recognized as saprophytic filamentous fungi found mainly in human-impacted areas and emerged as human pathogens in both immunocompetent and immunocompromised individuals. Last but not least, the final paper to be presented in this Special Issue attracts the attention of the reader to the fact that we live every day with and among filamentous fungi. Vaali et al. [18] aimed to establish an etiology-based connection between the symptoms experienced by the occupants of a workplace and the presence of toxic dampness microbiota in the building. Among the conclusions, the cytotoxicity test of the indoor air condensate is a promising tool for risk assessment in moisture-damaged buildings.

As a concluding remark, I, the Guest Editor, wish to thank all the authors and the reviewers for their significant contributions to this Special Issue and for making it a highly successful and timely collection of papers. Our acknowledgements also go to the whole MDPI team, i.e., assistant editors, editors, Editor-in-Chief, the production office, website management, etc.

## References

[1] Blackwell M. (2011). The fungi: 1, 2, 3 … 5.1 million species?. Am. J. Bot..

[2] Rodriguez R.J., White J.F., Arnold A.E., Redman A.R. (2009). Fungal endophytes: Diversity and functional roles. New Phytol..

[3] Verma A., Gupta P., Rai N., Tiwari R.K., Kumar A., Salvi P., Kamble S.C., Singh S.K., Gautam V. (2022). Assessment of biological activities of fungal endophytes derived bioactive compounds Isolated from *Amoora rohituka*. J. Fungi.

[4] Deshmukh S.K., Dufossé L., Chhipa H., Saxena S., Mahajan G.B., Gupta M.K. (2022). Fungal endophytes: A potential source of antibacterial compounds. J. Fungi.

[5] Nagarajan K., Ibrahim B., Ahmad Bawadikji A., Lim J.W., Tong W.Y., Leong C.R., Khaw K.Y., Tan W.N. (2021). Recent developments in metabolomics studies of endophytic fungi. J. Fungi.

[6] Meade E., Hehir S., Rowan N., Garvey M. (2022). Mycotherapy: Potential of Fungal Bioactives for the Treatment of Mental Health Disorders and Morbidities of Chronic Pain. J. Fungi.

[7] Sinaeve S., Husson C., Antoine M.-H., Welti S., Stévigny C., Nortier J. (2022). Nephroprotective Effects of Two Ganoderma Species Methanolic Extracts in an In Vitro Model of Cisplatin Induced Tubulotoxicity. J. Fungi.

[8] Rana S., Singh S.K., Dufossé L. (2022). Multigene Phylogeny, Beauvericin Production and Bioactive Potential of *Fusarium* Strains Isolated in India. J. Fungi.

[9] Kuvarina A.E., Rogozhin E.A., Sykonnikov M.A., Timofeeva A.V., Serebryakova M.V., Fedorova N.V., Kokaeva L.Y., Efimenko T.A., Georgieva M.L., Sadykova V.S. (2022). Isolation and Characterization of a Novel Hydrophobin, Sa-HFB1, with Antifungal Activity from an Alkaliphilic Fungus, *Sodiomyces alkalinus*. J. Fungi.

[10] Vivekanandhan P., Swathy K., Murugan A.C., Krutmuang P. (2022). Insecticidal Efficacy of *Metarhizium anisopliae* Derived Chemical Constituents against Disease-Vector Mosquitoes. J. Fungi.

[11] Tamizi A.-A., Mat-Amin N., Weaver J.A., Olumakaiye R.T., Akbar M.A., Jin S., Bunawan H., Alberti F. (2022). Genome Sequencing and Analysis of *Trichoderma* (Hypocreaceae) Isolates Exhibiting Antagonistic Activity against the Papaya Dieback Pathogen, *Erwinia mallotivora*. J. Fungi.

[12] Fu Z., Liu Y., Xu M., Yao X., Wang H., Zhang H. (2022). Absolute Configuration Determination of Two Diastereomeric Neovasifuranones A and B from *Fusarium oxysporum* R1 by a Combination of Mosher’s Method and Chiroptical Approach. J. Fungi.

[13] Mozūraitis R., Apšegaitė V., Radžiutė S., Aleknavičius D., Būdienė J., Stanevičienė R., Blažytė-Čereškienė L., Servienė E., Būda V. (2022). Volatiles Produced by Yeasts Related to *Prunus avium* and *P. cerasus* Fruits and Their Potentials to Modulate the Behaviour of the Pest *Rhagoletis cerasi* Fruit Flies. J. Fungi.

[14] He H., Yan Y., Dong D., Bao Y., Luo T., Chen Q., Wang J. (2022). Effect of *Issatchenkia terricola* WJL-G4 on Deacidification Characteristics and Antioxidant Activities of Red Raspberry Wine Processing. J. Fungi.

[15] El-Gendi H., Saleh A.K., Badierah R., Redwan E.M., El-Maradny Y.A., El-Fakharany E.M. (2022). A Comprehensive Insight into Fungal Enzymes: Structure, Classification, and Their Role in Mankind’s Challenges. J. Fungi.

[16] Derbyshire E. (2022). Fungal-Derived Mycoprotein and Health across the Lifespan: A Narrative Review. J. Fungi.

[17] Mello T.P., Barcellos I.C., Aor A.C., Branquinha M.H., Santos A.L.S. (2022). Extracellularly Released Molecules by the Multidrug-Resistant Fungal Pathogens Belonging to the *Scedosporium* Genus: An Overview Focused on Their Ecological Significance and Pathogenic Relevance. J. Fungi.

[18] Vaali K., Tuomela M., Mannerström M., Heinonen T., Tuuminen T. (2022). Toxic Indoor Air Is a Potential Risk of Causing Immuno Suppression and Morbidity—A Pilot Study. J. Fungi.

